# Small-molecule inhibition of pyruvate phosphate dikinase targeting the nucleotide binding site

**DOI:** 10.1371/journal.pone.0181139

**Published:** 2017-07-10

**Authors:** Alexander Minges, Georg Groth

**Affiliations:** Cluster of Excellence on Plant Sciences (CEPLAS), Institute of Biochemical Plant Physiology, Heinrich Heine University Düsseldorf, 40204 Düsseldorf, Germany; University of Pittsburgh School of Medicine, UNITED STATES

## Abstract

Pyruvate phosphate dikinase (PPDK) is an essential enzyme of C_4_ photosynthesis in plants, catalyzing the ATP-driven conversion of pyruvate to phosphoenolpyruvate (PEP). It is further used by some bacteria and unicellular protists in the reverse, ATP-forming direction. Many weed species use C_4_ photosynthesis in contrast to world’s major crops, which are C_3_ plants. Hence inhibitors of PPDK may be used as C_4_-specific herbicides. By screening a library of 80 commercially available kinase inhibitors, we identified compounds derived from bisindolylmaleimide (bisindolylmaleimide IV, *IC*_50_ = 0.76 ± 0.13 μM) and indirubin (indirubin-3’-monoxime, *IC*_50_ = 4.2 ± 0.9 μM) that showed high inhibitory potency towards PPDK and are among the most effective PPDK inhibitors described today. Physiological studies on leaf tissues of a C_4_ model plant confirmed *in vivo* inhibition of C_4_-driven photosynthesis by these substances. Moreover, comparative docking studies of non-inhibitory bisindolylmaleimide derivatives suggest that the selectivity towards PPDK may be increased by addition of functional groups to the core structure.

## Introduction

The main characteristic of C_4_ plants is their ability to thrive in warm and dry environmental conditions by efficient usage of nitrogen, water and CO_2_ [1–3]. This is ensured by spatial separation of the primary carbon fixation in mesophyll cells from CO_2_ release to the Calvin-Benson Cycle in the bundle sheet cell chloroplasts, leading to a much more efficient carbon fixation compared to C_3_ plants, where primary carbon fixation takes place directly in the Calvin-Benson Cycle [4].

Many of today’s crops, such as wheat or rice, use the C_3_ pathway, while most of the world’s worst weeds (e.g. *Cyperus rotundus* or *Echinochloa crus-galli*) are C_4_ plants [5]. Hence, an herbicide that specifically targets C_4_ plants would be of highest interest to ensure high crop yields in the context of increasing resistances against conventional herbicides. A potential target of the C_4_ photosynthetic pathway is pyruvate phosphate dikinase (PPDK) which is one of the rate limiting enzymes of C_4_ photosynthesis [6]. It catalyzes the ATP-driven interconversion of pyruvate to phosphoenolpyruvate (PEP) and hereby regenerates PEP that is used as the primary CO_2_ acceptor in C_4_ plants. PPDK is composed of three distinct domains with well-defined functionalities (all residue numbers according to *Flaveria trinervia* notation): An N-terminal nucleotide binding domain (NBD, aa 1-340), a central domain (CD, aa 381-516) and a C-terminal PEP/pyruvate binding domain (PBD, aa 534-874). The CD is linked to both substrate binding domains via two flexible linker regions (aa 341-380 and aa 517-533) and includes the catalytic His456 residue which is used in the transfer of a phosphoryl group from the nucleotide substrate ATP bound to the NBD to pyruvate at the PBD and vice versa. This phospho-transfer has to bridge a distance of approx. 40 Å from one substrate binding site to the other. Hence a swiveling domain mechanism was proposed to explain the large rotational and translational movement of the CD required for phosphoryl group transfer between the two catalytic centers [7, 8]. This swiveling mechanism has been supported by X-ray crystallographic data of PPDKs from *Clostridium symbiosum*, *Trypanosoma brucei* and *Zea mays*, which have resolved two extreme conformations of the CD—one facing the NBD, the other one facing the PBD [7, 9, 10]. A recently resolved structure of PPDK from the C_4_ plant *F. trinervia*, representing a conformational intermediate of the catalytic cycle, illustrates that the proposed CD swiveling motion proceeds via at least one discrete sub-step [11]. Thus, similar to other proteins employing large domain movements such as the F_1_-ATPase or the bacterial flagellar motor, PPDK also seems to operate discrete sub-steps in the movement of the CD associated with the catalytic cycle.

Catalytic activity of PPDK is regulated by phosphorylation of a threonine residue (aa 454 in *Flaveria*), located in close proximity to the catalytic histidine (aa 456 in *Flaveria*). Remarkably, both phosphorylation and dephosphorylation of the regulatory threonine are catalyzed by a bifunctional enzyme, the PPDK regulatory protein (PPDK-RP) [12–15]. In plants, activation and inactivation of PPDK by PPDK-RP are light-mediated and depend on ADP and AMP levels at dark and light periods. High levels of stromal ADP stimulate phosphorylation in the dark and at the same time inhibit dephosphorylation of the regulatory threonine [14, 16]. The three-dimensional structure of PPDK-RP from *Zea mays* has been recently solved, providing insights into the unusual bifunctionality of this protein [17].

While PPDK is essential for all C_4_ plants, it is not crucial for C_3_ plants: PPDK knock-out mutants of *Oryza sativa* and *Arabidopsis thaliana* grown under normal environmental conditions do not exhibit any obvious phenotypical anomalies [18, 19]. Moreover, although PPDK is used by some bacteria and unicellular parasitic protists such as *Giardia lamblia*, *Trichomonas vaginalis*, or *Entamoeba histolytica*, no homologue of PPDK is known in insects or vertebrates. This absence in higher animals makes PPDK an interesting target for antimicrobial and antiparasitic drugs as well as C_4_-specific herbicides.

Past studies have identified several substances originating from marine organisms with inhibitory effects on PPDK [20–23]. Among these, hydroxyquinones, ilimaquinone, ethylsmenoquinone and smenoquinone showed inhibitory constants in the higher micromolar range. The mode of action of these compounds is still not fully understood. In some cases, a mixed-type inhibition with regard to ATP was reported, which may hint at a binding at or near the nucleotide binding side. In a more recent study by Wu *et al.* [24], the nucleotide binding site was targeted directly by tight binding, space-filling flavone derivatives, resulting in a number of hits with high inhibitory potency and specificity towards PPDK.

Building on these results, the search of novel inhibitors of PPDK may not only focus on the ATP binding site as a primary—but often neglected as “generic”—target, but may also include known kinase inhibitors. In a similar attempt, Armstrong *et al.* [25] successfully identified inhibitors of carbohydrate sulfotransferase using a library of known kinase inhibitors. Here we report on the identification of novel high-potency inhibitors of PPDK targeting the nucleotide binding site from a set of commercially available kinase inhibitors by using PPDK from the C_4_ plant *Flaveria trinervia* in an *in vitro* assay. Further studies on leaf tissues of the C_4_ model plant maize demonstrate that these compounds inhibit C_4_-driven photosynthesis *in vivo* and confirm that these substances inhibit PPDK at naturally occuring enzyme and substrate concentrations.

## Materials and methods

### Heterologous expression and protein purification

Heterologous gene expression and purification of the recombinant PPDK were performed as described in [11]. A modified pET-16b vector (Merck, Darmstadt, GER) which contained an N-terminal histidine_10_ tag, followed by a TEV cleavage site and the sequence encoding for PPDK from *Flaveria trinervia* (EMBL X75516) was used for heterologous expression in *E. coli* strain BL21 (DE3). Transformed cells were grown to an *OD*_600_ of 0.6–0.8 in 2YT medium at 30°C before expression was induced by the addition of 0.1 mM isopropyl-*β*-D-1-thiogalactopyranoside. Cells were harvested after over night incubation at 30°C and 180 rpm. Preceding purification, cells were resuspended in lysis buffer (50 mM Tris/HCl pH 7.5, 300 mM NaCl, 10 mM imidazole, 10 mM MgSO_4_, 10% (w/v) glycerol, 5 mM DTT, 0.002% (w/v) phenylmethanesulfonylfluoride) and disrupted using a cell disruptor (Constant Systems). PPDK was purified using a nickel affinity chromatography column (GE Healthcare, Munich, GER). Purification (50 mM Tris/HCl pH 7.5, 300 mM NaCl, 10 mM MgSO_4_, 10% (w/v) glycerol, 5 mM DTT) and elution buffer (50 mM Tris/HCl pH 7.5, 300 mM NaCl, 500 mM imidazole, 10 mM MgSO_4_, 10% (w/v) glycerol, 5 mM DTT) were used for further purification steps. PPDK bound to the column was washed in steps of 50 mM, 150 mM and 200 mM imidazole, before final elution with 500 mM imidazole. Protein-rich fractions were pooled and a PD-10 desalting column (GE Healthcare) was used to change the elution buffer for assay buffer (100 mM Tris/HCl pH 8.0, 10 mM MgCl_2_, 2.5 mM KH_2_PO_4_, 6 mM glucose-6-phosphate, 5 mM NaHCO_3_, 0.1 mM EDTA, 5 mM DTT). The buffer-exchanged sample was eventually concentrated by ultrafiltration (30 kDa cutoff, Millipore).

### PPDK activity assay and inhibitor screening

A set of 80 compounds from a commercially available kinase inhibitor library (#10505; Cayman Chemicals, Ann Arbor, MI, USA) was screened for its effects on *F. trinervia* PPDK. Activity of purified PPDK was measured according to Salahas *et al.* [26], Doyle *et al.* [20] in a 96 well microtiter plate layout by a coupled spectrophotometrical assay. In this assay, the carboxylation of PEP by PEPC is linked to the oxidation of NADH by NADH-malate dehydrogenase (NADH-MDH). PEP is formed by PPDK via ATP-driven phosphorylation of pyruvate. Eventually, the consumption of one molecule NADH is equivalent to the formation of one molecule of PEP by PPDK. The assay was performed in a sample volume of 100 μL at 30°C in a M200 plate reader (Tecan, Crailsheim, GER). 0.2 μM PPDK were mixed with 0.2 mM NADH, 2.5 mM sodium pyruvate, 0.8 U bacterial PEPC (Sigma-Aldrich, Darmstadt, GER) and 2 U NADH-MDH (Sigma-Aldrich). The reaction mixture was filled up to a total volume of 100 μL per sample with assay buffer (100 mM Tris/HCl pH 8.0, 10 mM MgCl_2_, 2.5 mM KH_2_PO_4_, 6 mM glucose-6-phosphate, 5 mM NaHCO_3_, 0.1 mM EDTA, 5 mM DTT). PPDK was pre-incubated for 20 min at 30°C to ensure full activity. Inhibitors were dissolved in water free dimethyl sulfoxide (DMSO) with a final concentration of 10 mM and were added prior to adjusting the final sample volume. To account for possible effects of DMSO on PPDK activity, the solvent was added to the controls in an amount equal to the largest DMSO concentration used in the experiments. Incubation time of the final sample mixture including inhibitors and PPDK was 15 min. Initial comparative screening was perfomed using 100 μM of each compound. The absorbance at 340 nm was recorded for 30 s with an interval of 300 ms. The reaction was started by automated injection of 1.25 mM ATP. The activity was then deduced from the initial slope, discarding the first 10 s due to noise caused by mixing effects and normalized to the DMSO-treated controls. Six compounds with the highest potency were chosen for further analysis of their inhibitory potential. Since compounds of the bisindolylmaleimide class showed similar effects on PPDK activity, only two of them (BIM IV and Go6983) were chosen as representatives of this class. The half maximal (50%) inhibitory concentration *IC*_50_ of the selected compounds was calculated by measuring the activity as described before for at least ten data points in the concentration range of 0 μM to 200 μM of the inhibitory compound in at least triplicates. A four-parameter log-logistic dose-response curve was then globally fitted to the individual replicates using the R software collection [27] and the R package “drc” [28] as proposed by assessment of Akaike’s information criterion (AIC) [29, 30]. PPDK was incubated for 10 min with the inhibitors before each measurement. To exclude PEPC or NADH-MDH inhibition by the selected compounds, the initial screening assay was repeated in triplicates for those compounds without adding PPDK and starting the reaction by injection of PEP instead of ATP. Determined activities were compared to the control and statistically analyzed using a paired t-test.

### Inhibition of oxygen evolution during C_4_ photosynthesis

Oxygen (O_2_) evolution, driven by the C_4_ acid cycle, was measured according to *refs* [21, 22, 31]. The reaction chamber of a Clark-type O_2_ electrode (Hansatech, Norfolk, UK) was filled with 1 mL of degassed buffer containing 0.33 M sorbitol, 2.5 mM NaH_2_PO_4_, 2.5 mM MgCl_2_, 25 mM HEPES/KOH pH 7.5, 50 μM MnCl_2_ and 2.5 mM dithiothreitol (DTT). An area sized approx. 1 cm^2^ was cut from a mature leaf of the C_4_ model plant *Zea mays* and processed into slices of ∼ 1 mm width. The leaf slices were added to the electrode chamber and the recording of O_2_ evolution was started while keeping the chamber in the dark. The O_2_ evolution rate recorded in this period was later substracted from the subsequently recorded rates to account for the electrode drift. Once the system had stabilized, the chamber was illuminated using a Schott KL 1500 electronic light source. Again, the system was given time to stabilize before the C_4_-driven O_2_ evolution was initiated by the addition of first NaHCO_3_ and then pyruvate in final concentrations of 4 mM each. In agreement with [21], the NaHCO_3_-dependent O_2_ evolution rate was negligible. The O_2_ evolution rate after adding pyruvate was recorded for at least 3 min after it had stabilized and was used as the control rate. Then a total amount of 20 μg and 40 μg (if possible due to solubility limit) of the test substance dissolved in DMSO was added to the reaction chamber and the O_2_ evolution rate was again recorded for at least 3 min. All measurements were done in triplicates.

Directly following the measurement, leaf slices were removed from the chamber and transferred to 1 mL of 80% acetone (v/v) for chlorophyll extraction. The samples were kept at 4°C in the dark for 48 h. The total chlorophyll amount was determined using a DU800 (Beckman Coulter, USA) spectrophotometer and calculated using Eq (1) [32].

$$\begin{array}{c} \text{total}\;\text{chl}\;\left[\mu \text{g}\;{\text{mL}}^{-1}\right]=\left(20.2\times OD_{645}\right)+\left(8.02\times OD_{663}\right) \end{array}$$ (1)

The O_2_ evolution rates in the presence of the inhibitors were compared to the respective controls and statistically analyzed using a two-sided t-test. Relative inhibition of O_2_ evolution was expressed in percent and calculated as stated in Eq (2).

$$\begin{array}{c} \text{inhibition}\;\left[\%\right]=100-\frac{\text{O}{}_{2}\;\text{evolution}\;\text{treated}\times 100}{\text{O}{}_{2}\;\text{evolution}\;\text{control}} \end{array}$$ (2)

### Docking of compounds into the nucleotide binding site

Since all of the most-inhibiting compounds are described as inhibitors for nucleotides or nucleic acids in their original targets, virtual docking of these molecules was performed into the ATP binding cleft of PDB 5JVL chain D. The bounding box was sized 20 Å in each dimension and centered at the position of the 4’-carbon atom of the nucleotide analogue 2’-Br-dAppNHp bound to this structure. 2’-Br-dAppNHp was removed after filling missing sidechains using a rotamer library [33] and protonation with tools from UCSF Chimera [34]. A maximum of 200 conformers of each compound was generated using RDKit [35]. Conformers with RMSDs below 0.1 Å to other conformers in the same set were discarded. Those sets of conformers were docked with the AutoDock Vina-derived program smina [36, 37]. The docked results were then ranked according to their predicted binding energy. Images were prepared using PyMOL [38]

## Results and discussion

**Fig 1:**
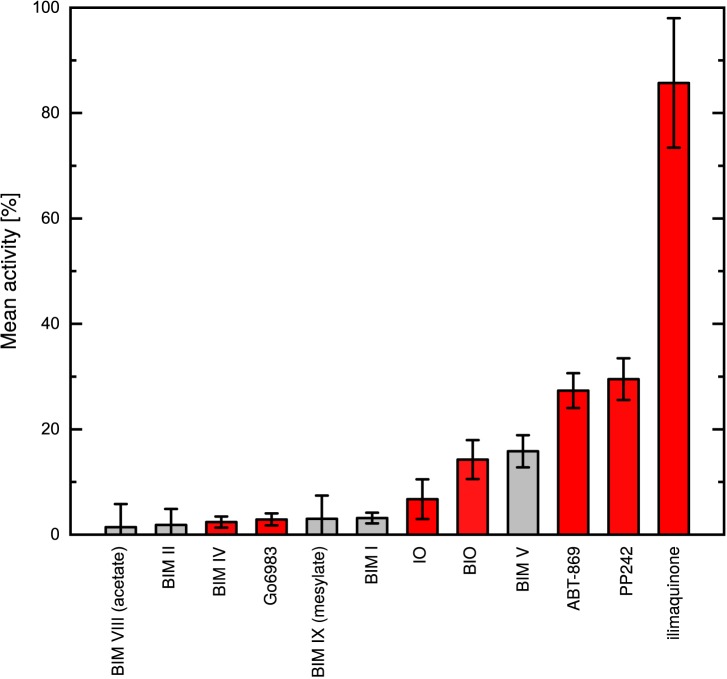
Mean activity of PPDK at inhibitor concentrations of 100 μM. Depicted from left to right are the most potent inhibitors identified in the initial screening assay with the known PPDK inhibitor ilimaquinone. Inhibitors colored red were further analyzed for their *IC*_50_ values. Errors shown are standard deviations (SD).

To search for inhibitory compounds blocking the nucleotide binding site, a set of 80 commercially available kinase inhibitors was screened for effects on PPDK from the C_4_ plant *Flaveria trinervia*. Analysis of the initial test set by plotting the residual PPDK activity after inhibitor treatment revealed several promising candidates. Of the 80 tested compounds, 22 led to 50% inhibition of PPDK activity at a concentration of 100 μM. Eleven of the tested compounds reduced the activity even to levels below 30%. Seven of the top scoring compounds belong to the chemical class of bisindolylmaleimides which are characterized by an indol-substituted maleimide structure and varying other structural elements. Six of the bisindolylmaleimide compounds caused an almost complete loss of PPDK activity when applied at concentrations of 100 μM. Residual activity measured was in the range of 1–3% of the DMSO-treated controls except for bisindolylmaleimide V showing a residual activity of 15% (Fig 1). Compounds of the bisindolylmaleimide class have been previously identified as high-pontency inhibitors of human protein kinase C (PKC) with *IC*_50_ values in the nanomolar range [39–42]. A second class of PPDK inhibitors is formed by indirubin-3’-monoxime (IO) and its bromized derivative 6-bromoindirubin-3’-monoxime (BIO), which are described as ATP-competitive inhibitors of cyclin-dependent kinases (CDK, *IC*_50_: 50–100 nM) and glycogen synthase kinase 3*β* (GSK3*β*, *IC*_50_: 5–50 nM) [43, 44]. However, residual activities observed after treatment with this chemical class were up to two-fold higher than with the bisindolylmaleimides. The other remaining compounds identified in our screening reducing PPDK activity below 30% (Fig 1) – ABT-869 and PP242 – are not structurally related to each other or to the two other groups. Both are ATP-competitive inhibitors, targeting receptor tyrosine kinases (RYK, ABT-869) and mammalian target of rapamycin (mTOR, PP242) with *IC*_50_ values of 4 nM and 8 nM, respectively [45, 46].

To exclude the possibility that the inhibitors identified in our screening target PEPC or NADH-MDH and thereby bias the coupled enzymatic assay, the assay was repeated in the absence of PPDK and ATP and the reaction was started by the addition of PEP. Hence, only inhibitory effects on either PEPC or NADH-MDH were addressed in this experimental setup. The results were compared with those of the controls and statistically analyzed. Only for BIO, a significant inhibition of PEPC or NADH-MDH was apparent (data not shown). The remaining activity was still well above 60% of the control. Therefore it is unlikely that the drop of PPDK activity observed for BIO to about 15% is caused by PEPC or NADH-MDH inhibition alone, but is mainly caused by inhibition of PPDK. For all other compounds, no significant inhibition of PEPC or NADH-MDH was detected.

**Fig 2:**
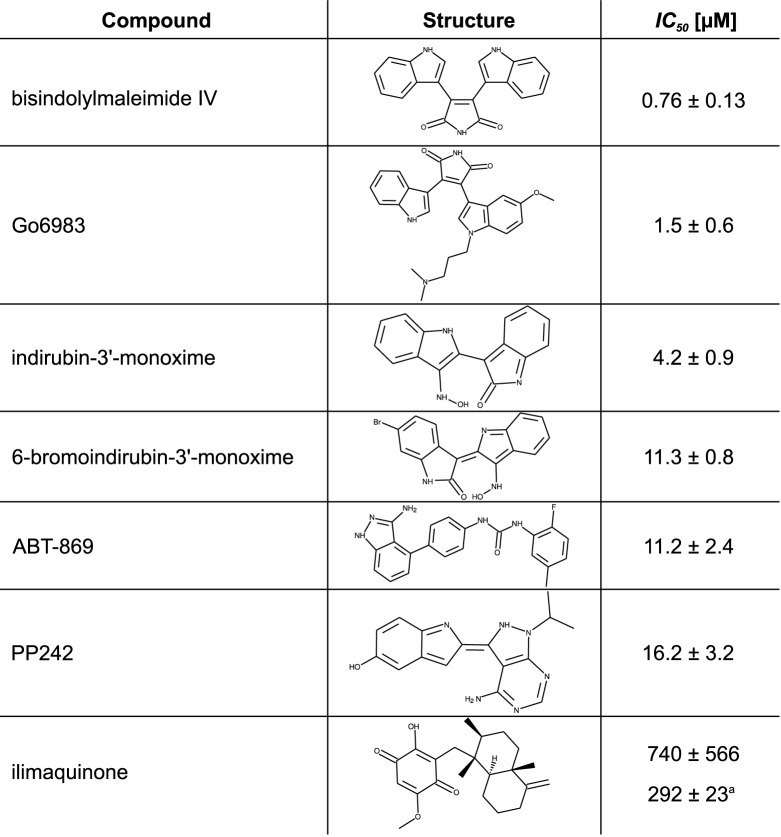
Structures and *IC*_50_ values of PPDK inhibitors. ^a^ *IC*_50_ of ilimaquinone measured for *Zea mays* PPDK from leaf extracts according to Haines *et al.* [21]. Errors shown are standard errors (SE).

**Fig 3:**
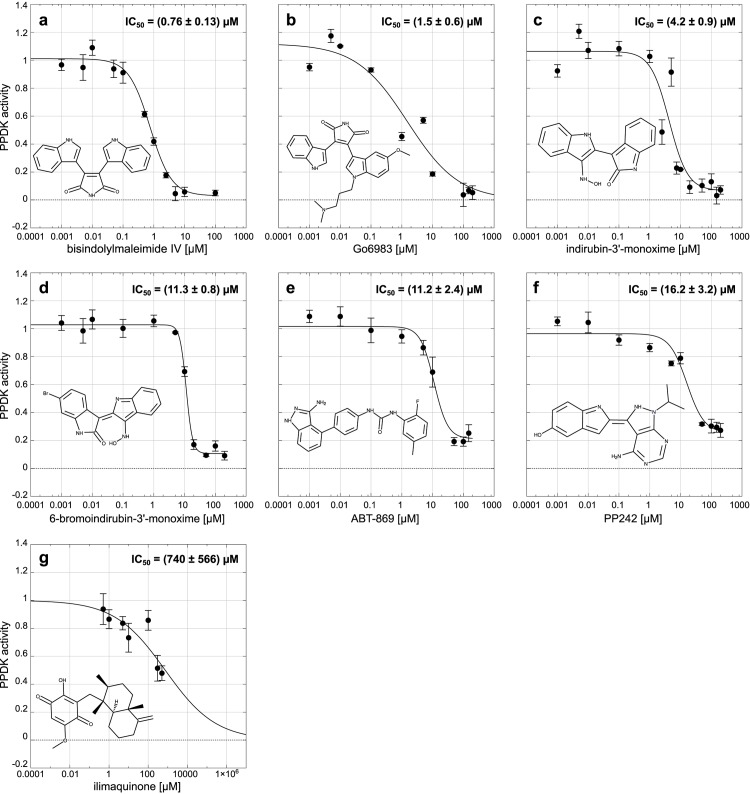
Dose-response curves of selected inhibitors of C_4_ plant PPDK. The PPDK concentration is kept constant at 0.2 μM, while the inhibitor concentrations are varied between 0 μM and 200 μM. The mean activity from three experiments relative to the control is plotted for bisindolylmaleimide IV (a), Go6983 (b), indirubin-3’-monoxime (c), 6-bromoindirubin-3’-monoxime (d), ABT-869 (e), PP242 (f) and the PPDK specific inhibitor ilimaquinone (g). The *IC*_50_ values were calculated from these data by non-linear regression using log-logistic dose-response functions. Errors shown are standard errors of the mean (SEM).

The *IC*_50_ of the two indirubines, ABT-869, PP242, bisindolylmaleimide IV (BIM IV) and Go6983 were experimentally determined (Fig 2). For comparison, the PPDK specific inhibitor ilimaquinone [21] was added to the test set. In accordance to the measured activities from the initial screening, the two bisindolylmaleimides, BIM IV and Go6983, performed best with *IC*_50_ values (mean ± standard error) of 0.76 ± 0.13 μM and 1.5 ± 0.6 μM, respectively. An about tenfold lower inhibitory potency was shown by the two indirubines. The *IC*_50_ of IO was determined at 4.2 ± 0.9 μM. The bromized form showed an even higher value of 11.3 ± 0.8 μM. The *IC*_50_ of ABT-869 and PP242 were more than 10-fold higher than those of the two bisindolylmaleimides with 11.2 ± 0.24 μM and 16.2 ± 0.32 μM. The PPDK-specific inhibitor ilimaquinone was the least potent inhibitor in this series with an *IC*_50_ of 740 ± 566 μM (Fig 3). In total, the inhibitory potency of the bisindolylmaleimides identified in this study is comparable and in case of BIM IV even higher than for the alkyl-substituted flavonoids previously described by Wu *et al.* [24].

A primary obstacle for postemergent herbicides clearly is their uptake into the plant, particularly penetration of the leaf cuticle [47]. Consequently, the exact formulation of adjuvants to promote leaf penetration is one of the major issues in herbicide research and development. However, once inside the leaf, the extensive network of plasmodesmata in the bundle sheet cells of C_4_ plants faciliates further spreading of the applied chemical. In leaf slice assays, the cuticular barrier is bypassed by direct exposure of parts of the plasmodesmata to the surrounding buffer while keeping the integrity of the intra- and intercellular C_4_ photosynthetic apparatus intact. This allows to study the effect of inhibitors in a native cellular milieu. To substantiate the results of our *in vitro* assay and to elaborate the effect of the compounds identified in this assay under *in vivo* conditions, we thus applied oxgen measurements on isolated leaf slices of the C_4_ model plant maize.

**Fig 4:**
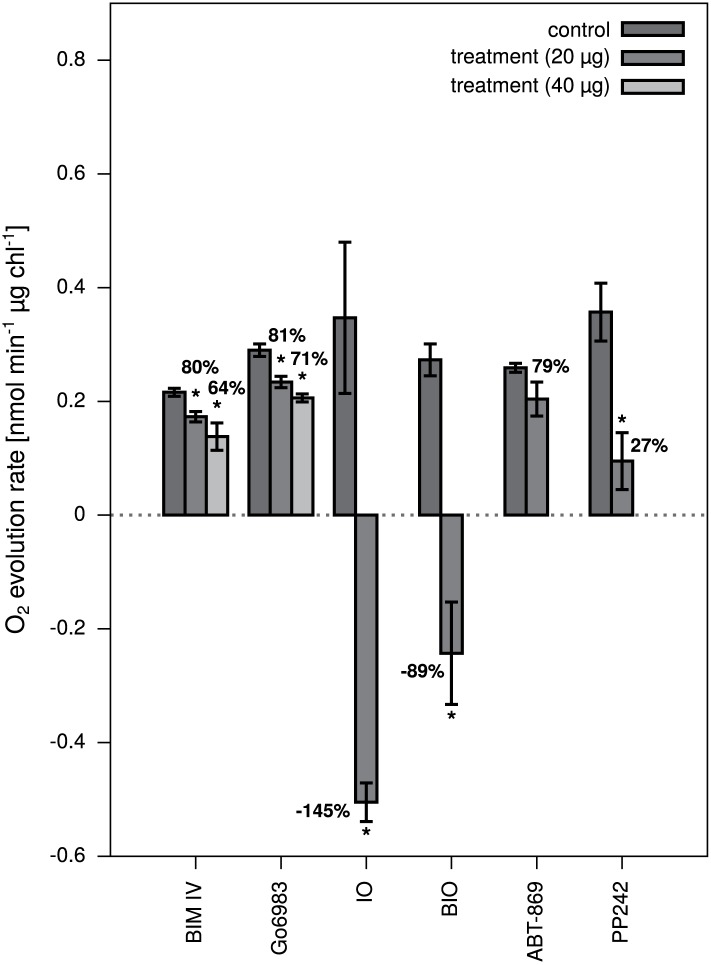
Oxygen evolution *in vivo* in the presence of putative PPDK inhibitors. The rate of O_2_ evolution of *Zea mays* leaf slices was measured in solution with a Clark-type electrode. PPDK inhibitors identified in the *in vitro* screening assay were added to the leaf slices in a final concentration of 20 μg mL^−1^. A significant decrease (*p* ≤ 0.05) was observed for BIM IV, IO, BIO and PP242. Rates were normalized to the total amount of chlorophyll in the leaf samples and corrected for electrode drift. O_2_ evolution rates relative to the respective controls are given in percent. Noteworthy, the O_2_ evolution rate in the presence of IO and BIO was negative, hence representing a consumption of O_2_. Errors shown are SEM.

Of the six putative PPDK inhibitors characterized in the *in vitro* assay, five led to a significant decrease in C_4_-dependent O_2_ evolution rate (Fig 4). Two of them (IO and BIO) even show negative rates, representing a dramatic net O_2_ consumption. However, both compounds include an oxime group structure, known to scavange molecular oxygen from aqeous solutions [48]. Thus, the O_2_ consumption observed with IO and BIO is probably related to a chemical rather than a biological effect.

For the remaining three inhibitors with significant effects on the O_2_ evolution rate, BIM IV inhibits O_2_ evolution by 20% when applied at a final concentration of 20 μg mL^−1^ and 36% at 40 μg mL^−1^. Similarly, the inhibition observed for Go6983 is 19% at 20 μg mL^−1^ and 29% at 40 μg mL^−1^. Due to its solubility limit, PP242 was only applied at 20 μg mL^−1^. However, already at this concentration, 73% inhibition of photosynthetic oxygen evolution was observed (Fig 4).

**Fig 5:**
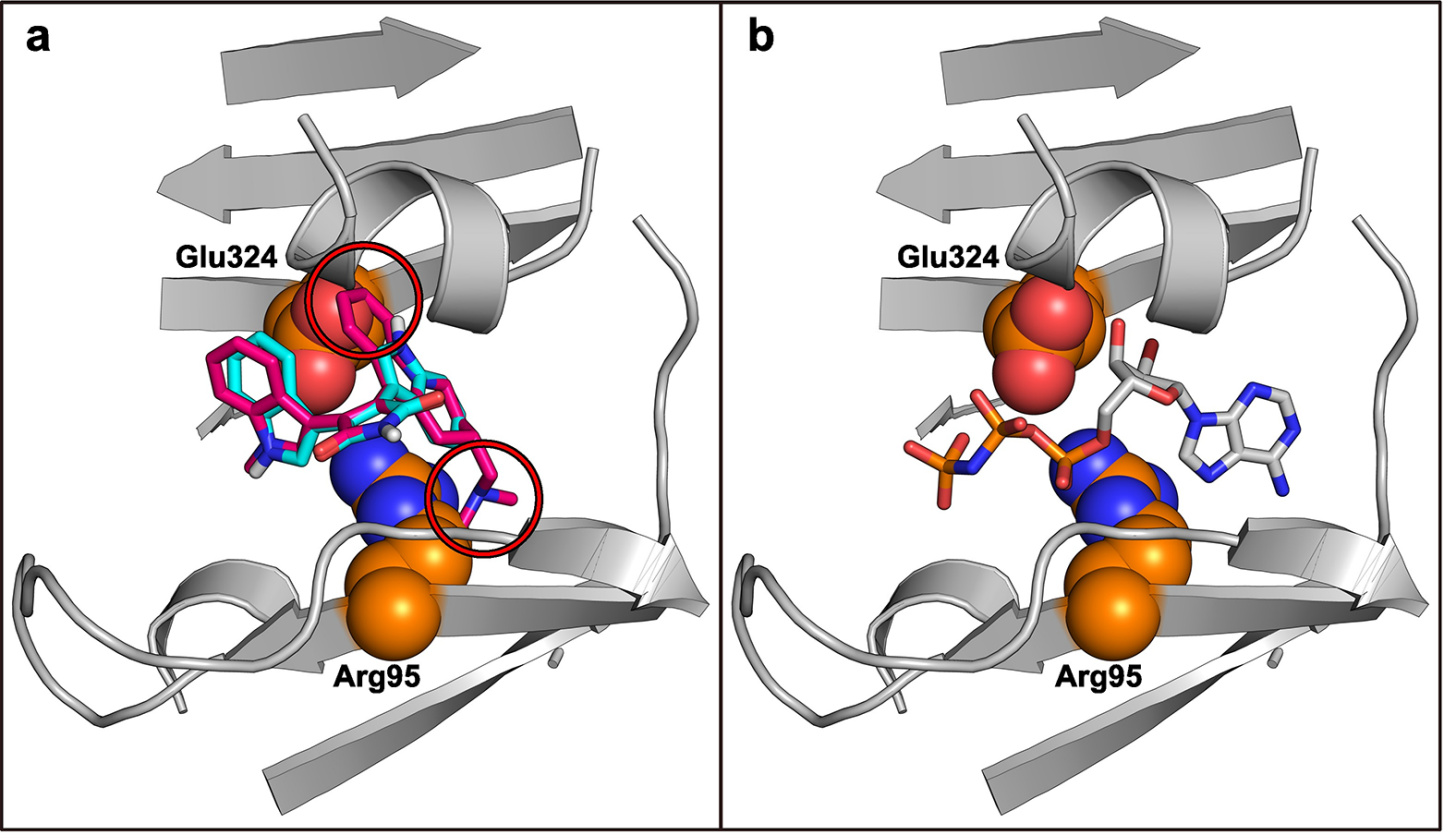
Predicted binding position of inhibitory and non-inhibitory bisindolylmaleimides at the nucleotide binding site of C_4_ plant PPDK. (a) Bisindolylmaleimide IV (cyan) docked to the nucleotide binding domain of PPDK (PDB 5JVL chain D). Only parts of the whole structure are shown for clarity. Almost identical binding poses are among the top-rated docking results of all bisindolylmaleimides tested and proven to be effective in this study. The structure of the related but non-inhibitory compound bisindolylmaleimide XI is shown in magenta. Severe clashes of this compound occur with residues Arg95 and Glu324 and are highlighted by red circles. (b) Bound nucleotide analogue 2’-Br-dAppNHp in 5JVL chain D.

Remarkably, although the bisindolylmaleimides seem to represent the best inhibitors tested in this study, one representative of this family (bisindolylmaleimide XI, BIM XI) did not show any effect on PPDK activity at all. In contrast to other bisindolylmaleimides, this compound contains a rather bulky cyclo-hexyl structure attached at one of its indole groups. This bulky extention may lead to steric clashes between the compound and the amino acid residues forming the nucleotide binding pocket. To test this hypothesis, an *in silico* docking approach was taken, and the eleven initial top scoring compounds, including the seven most effective bisindolylmaleimides, were docked into the nucleotide binding site of the high resolution PPDK structure of *F. trinervia* (PDB 5JVL chain D) [11]. Predicted binding energies for the bisindolylmaleimides were in the range of -10.5 Kcal mol^−1^ to -8.0 Kcal mol^−1^ thus reflecting a relatively tight binding which is in accordance with our activity assays. In addition, similar docking poses of all bisindolylmaleimides in regard to their core structural features were identified among the top-rated docking poses. Superimposition of BIM XI with those consensus poses revealed severe clashes with the surrounding protein structure at the highly-conserved residues Arg95 and Glu324 (Fig 5a), confirming our hypothesis that clashes resulting in unfavorable interactions with the surrounding binding pocket prevent inhibition of PPDK in case of BIM XI. Analysis of the influence of Arg95 and Glu324 on BIM XI binding by substitution mutagenesis is hampered as both residues are directly involved in ATP substrate binding. Hence, a mutation of these residues is likely to abolish PPDK activity. Additional structural information on the exact binding mode of bisindolylmaleimides by means of crystal structures in the presence of these compounds are probably needed to answer this question. In their original target PKC, bisindolylmaleimides are known to bind at the same position as the natural substrate ATP [49, 50]. Similarly, our docking results suggest that the binding mode of bisindolylmaleimides in PPDK largely overlaps with the bound nucleotide analogue in PPDK structure 5JVL [11] (Fig 5b).

In summary, we were able to identify novel high-potency inhibitors of PPDK by screening a kinase inhibitor library. The novel inhibitors, which belong to the chemical classes of bisindolylmaleimides and indirubins, are among the most effective inhibitors of PPDK, with BIM IV as the single most potent PPDK inhibitor identified today. The compounds have been previously described as specific inhibitors of protein kinase C (PKC) and other kinases involved in cancer development. The herbicidal potential of the novel PPDK inhibitors is substantiated by *in vivo* plant studies, which show significant inhibition of C_4_-dependent O_2_ evolution by BIM IV, Go6983 and PP242. Their relatively high potency with *IC*_50_ values in the higher nanomolar to lower micromolar range suggests that these compounds are interesting lead structures for further adaption to the PPDK nucleotide binding pocket. The fact that the extent and selectivity of PPDK inhibition indeed can be adapted by substitutions at the bisindolylmaleimide core is reflected by the observation that BIM XI shows no effect on PPDK activity due to potential steric clashes with amino acid side chains in the nucleotide binding pocket of PPDK. Because the newly identified PPDK inhibitors were originally designed for other kinases such as the PKC, they do not exclusively target PPDK in their current structure and chemical composition. However, this may be addressed in further studies by evolving bisindolylmaleimides towards tighter and more specific interaction with the PPDK’s nucleotide binding pocket while preserving their unprecedented inhibitory potential. Similar strategies have been successfully pursued in the past in the development of selective protein kinase inhibitors such as AMG706 for vascular endothelial growth factor receptor (VEGFR), PF-562271 for focal adhesion kinase (FAK) or GSK461364A for Polo-like kinase (PLK1) [51].
